# Qualitative characterizations of relationships among South African adolescent girls and young women and male partners: implications for engagement across HIV self‐testing and pre‐exposure prophylaxis prevention cascades

**DOI:** 10.1002/jia2.25521

**Published:** 2020-06-30

**Authors:** Leah E Holmes, Michelle R Kaufman, Albert Casella, Mutsa Mudavanhu, Lillian Mutunga, Tara Polzer, Jean Bassett, Annelies Van Rie, Sheree Schwartz

**Affiliations:** ^1^ Department of Epidemiology Johns Hopkins Bloomberg School of Public Health Baltimore MD USA; ^2^ Department of Health. Behavior & Society Johns Hopkins Bloomberg School of Public Health Baltimore MD USA; ^3^ Witkoppen Health and Welfare Clinic Johannesburg South Africa; ^4^ Social Surveys Africa Johannesburg South Africa; ^5^ Department of Epidemiology and Social Medicine University of Antwerp Antwerp Belgium

**Keywords:** HIV prevention cascade, pre‐exposure prophylaxis, HIV self‐testing, South Africa, adolescent girls and young women, relationships

## Abstract

**Introduction:**

Adolescent girls and young women (AGYW) in sub‐Saharan Africa have emerged as a priority population in need of HIV prevention interventions. Secondary distribution of home‐based HIV self‐test kits by AGYW to male partners (MP) is a novel prevention strategy that complements pre‐exposure prophylaxis (PrEP), a female‐controlled prevention intervention. The objective of this analysis was to qualitatively operationalize two HIV prevention cascades through the lens of relationship dynamics for secondary distribution of HIV self‐tests to MP and PrEP for AGYW.

**Methods:**

From April 2018 to December 2018, 2200 HIV‐negative AGYW aged 16‐24 years were enrolled into an HIV prevention intervention which involved secondary distribution of self‐tests to MP and PrEP for AGYW; of these women, 91 participants or MP were sampled for in‐depth interviews based on their degree of completion of the two HIV prevention cascades. A grounded theory approach was used to characterize participants’ relationship profiles, which were mapped to participants’ engagement with the interventions.

**Results:**

In cases where AGYW had a MP with multiple partners, AGYW perceived both interventions as inviting distrust into the relationship and insinuating non‐monogamy. Many chose not to accept either intervention, while others accepted and attempted to deliver the self‐test kit but received a negative reaction from their MP. In the few cases where AGYW held multiple partnerships, both interventions were viewed as mechanisms for protecting one’s health, and these AGYW exhibited confidence in accepting and delivering the self‐test kits and initiating PrEP. Women who indicated intimate partner violence experiences chose not to accept either intervention because they feared it would elicit a violent reaction from their MP. For AGYW in relationships described as committed and emotionally open, self‐test kit delivery was completed with ease, but PrEP was viewed as unnecessary. MP experience with the cascade corroborated AGYW perspectives and demonstrated how men can perceive female‐initiated HIV prevention options as beneficial for AGYW and a threat to MP masculinity.

**Conclusions:**

Screening to identify AGYW relationship dynamics can support tailoring prevention services to relationship‐driven barriers and facilitators. HIV prevention counseling for AGYW should address relationship goals or partner’s influence, and engage with MP around female‐controlled prevention interventions.

## 1 INTRODUCTION

Adolescent girls and young women (AGYW) aged 15‐24 years have emerged as a priority population in need of urgent intervention due to high HIV incidence rates in sub‐Saharan Africa and disproportionate gender differences in HIV risk [1, 2, 3]. Within South Africa, the interplay of relationship dynamics, schemas of masculine dominance and high prevalence of intimate partner violence (IPV) have strong implications for HIV prevention behaviours among young women and men [4, 5, 6, 7, 8, 9]. Despite recent increases in HIV testing among South Africans, many AGYW remain unaware of their own and their partners’ status, which presents barriers to successful HIV prevention [10, 11, 12, 13, 14]. Furthermore, men lag in engagement in HIV treatment and prevention cascades [10, 11, 12, 13, 14].

HIV self‐testing is increasing and offers opportunities for reaching populations less traditionally served by clinic‐based services, including men [10, 11, 12, 13, 14]. The use of secondary distribution of self‐test kits by AGYW to increase testing and status disclosure among male partners (MP) is a novel HIV prevention strategy [10, 11, 12, 13, 14]. One study recently demonstrated the effectiveness of this approach in antenatal care (ANC) and postnatal care settings [10] but may produce different results outside of the ANC context [10].

Pre‐exposure prophylaxis (PrEP), when taken consistently, is currently the female‐controlled prevention intervention with the greatest efficacy, and it has the potential to empower AGYW who are in relationships characterized by power imbalances [15]. Overall reductions in HIV acquisition in men and women have been demonstrated through randomized control trials of PrEP across multiple settings [15, 16, 17, 18, 19, 20, 21, 22]. However, PrEP effectiveness has not been consistently achieved across studies, particularly among young women outside of serodiscordant relationships, due to limited product adherence [15]. Still, the promise of PrEP for AGYW is great, should interventions succeed in generating demand, motivating uptake and supporting adherence [23].

There is limited evidence around how relationship‐level barriers and facilitators influence secondary distribution of HIV self‐tests and PrEP uptake and adherence among AGYW. Thus, this study applied a qualitative approach that operationalizes and evaluates how relationship landscapes influence two HIV prevention cascades: (1) secondary distribution of self‐test kits by AGYW to MPs; and (2) PrEP for AGYW. By sampling AGYW who did/did not complete each step of the HIV prevention cascades, we hope to generate a nuanced understanding of the interpersonal circumstances that empower or hinder women to take control of their sexual health. Based on our findings, we propose recommendations for implementation of relationship‐centred HIV interventions for AGYW in South Africa.

## 2 METHODS

### 2.1 Study population and intervention

In the parent study, the interventions offered were part of a DREAMS Innovations Challenge package designed to keep HIV‐negative AGYW in South Africa HIV‐free [24]. Between April 2018 and December 2019, 2200 AGYW in northern Johannesburg were screened for enrollment into the parent study. The intervention package contained two oral HIV self‐test kits (one for the AGYW and one for her MP to facilitate couples’ testing when preferred or for multiple MP as appropriate), an instructional pamphlet, a video explaining the self‐testing process, condoms and lubricant. Formative research was conducted beforehand to inform the intervention content and video messaging (Tembo, unpublished work).

AGYW were eligible for enrollment into the parent study if they were aged 16‐24 years, tested HIV negative, had a current male sexual partner for ≥3 months at enrollment, reported they were unaware of their partner’s HIV status, and did not report relationship violence or fear of violence. Participants were recruited at a primary health clinic or within their community through a mobile team. Study team members administered baseline surveys to AGYW, counselled AGYW on test kit delivery to MP, and offered PrEP as an additional prevention option. Following enrollment and receipt of the self‐tests, AGYW were contacted two weeks later to ascertain the self‐testing results and were reminded of PrEP as an additional prevention option. Follow‐up calls or clinic meetings continued for three months to monitor final outcomes.

Using predominantly follow‐up data, study staff selected AGYW to participate in a qualitative sub‐study assessing barriers and facilitators to completion of the MP testing and PrEP prevention cascades; AGYW who were eligible for the parent study but refused participation were also enrolled. AGYW were evenly recruited across each of the cascade steps, and MPs were invited to participate if their AGYW had provided consent for the study team to contact him. Participants were not recruited to be representative of parent study outcomes, but to inform future intervention adaptations.

Of note, DREAMS programming in the area focused exclusively on clinic and community‐based HIV testing and linkage to care for HIV‐positive cases. The DREAMS Innovations team accompanied the program at times to recruit AGYW testing HIV negative for the parent study.

### 2.2 Data collection and analysis

**Figure 1:**
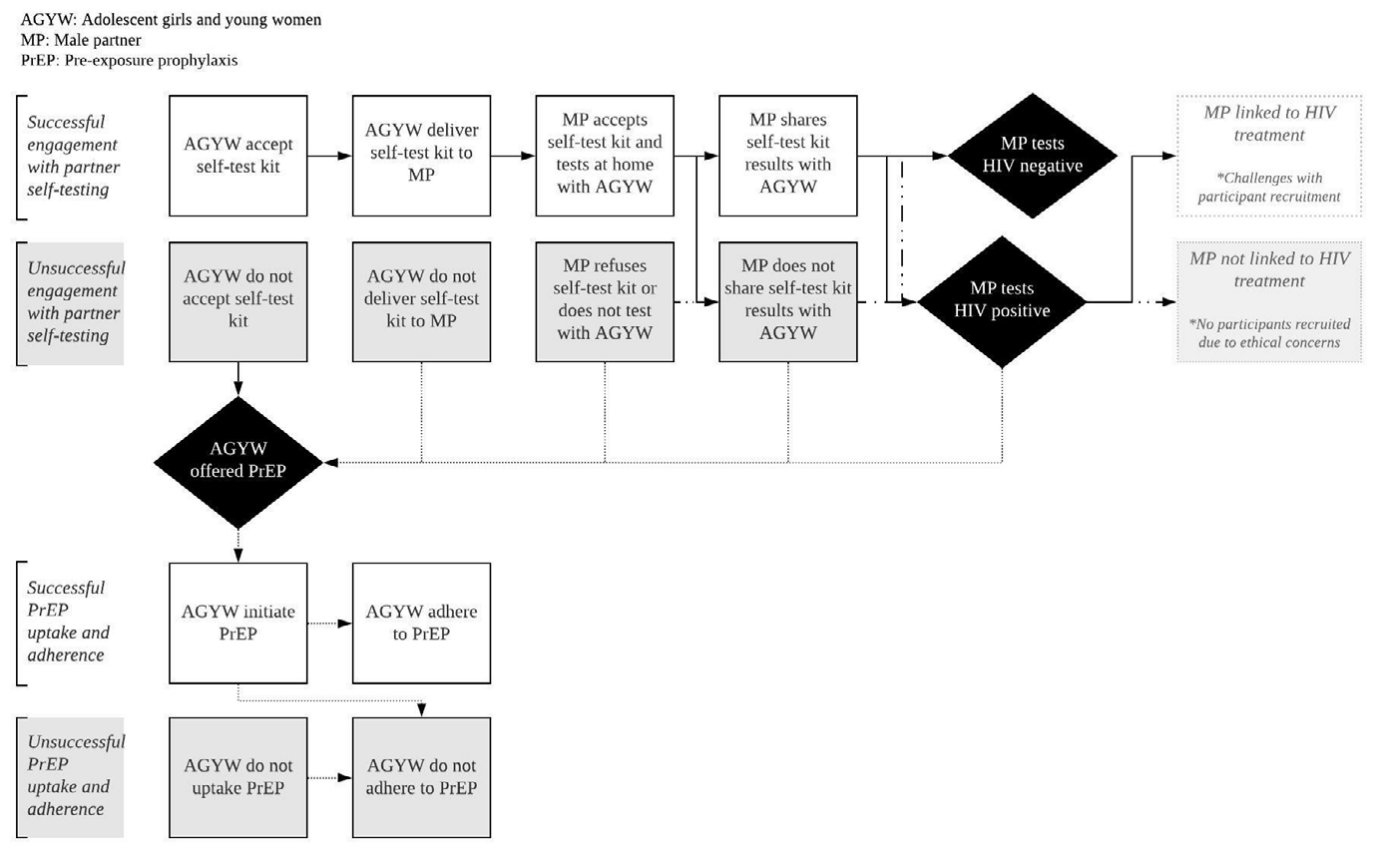
HIV prevention cascade for self‐testing and PrEP interventions. AGYW, adolescent girls and young women; MP, male partner; PrEP, pre‐exposure prophylaxis.

Qualitative, semi‐structured in‐depth interviews (IDIs) were conducted from May 2018 to February 2019. IDI participants (n = 50 AGYW parent study participants, 32 MPs, and 9 AGYW who declined the intervention), were purposively sampled based on the degree to which they completed the HIV prevention cascades (Figure 1). AGYW aged 18‐24 provided written informed consent, and AGYW <18 years provided assent and written parental consent. Qualitative study participants were compensated R100 ($7USD) for IDI transport. Permission to recruit MP refusing testing was not granted, so this perspective was only captured among AGYW. IDIs were completed with either the AGYW or MP in a relationship, but not both.

IDIs were conducted by four South African qualitative interviewers, exploring facilitators and barriers to completing the HIV prevention cascades. IDIs lasted approximately 35‐50 minutes and were conducted in English and/or local languages. IDIs were audio‐recorded, transcribed verbatim and translated into English. Weekly team meetings were held to debrief and modify probing to explore emerging themes. IDIs were conducted until saturation of themes was achieved.

Investigators employed an inductive, grounded theory approach to iteratively develop a codebook. Double coding of 30 randomly selected transcripts was performed with an *a priori* agreement level of 85% (below which full double coding would have been applied). Post‐coding analyses employed a deductive approach that mapped participants’ engagement with the self‐testing and PrEP cascades to the relationship profiles that emerged.

Ethical approval was granted by the Human Research Ethics Committee at the University of Witwatersrand in Johannesburg, South Africa, and oversight seconded by the Johns Hopkins Bloomberg School of Public Health to the South African Committee.

### 2.3 Context from the parent study

In the parent study, acceptability of HIV self‐testing was high, with over 95% of AGYW accepting and delivering self‐test kits to their partners, and over 80% of MP who were offered the test accepting and showing the results to their female partners. Over a third of AGYW were interested in PrEP, but only 3% initiated and even fewer adhered to PrEP.

## 3 RESULTS

Demographic and relationship characteristics of the 91 IDI participants are detailed in Table 1. Over half of the participants were in steady (non‐casual) relationships, but not living with their partner. Over 30% of AGYW believed their MP had other sexual partners.

**Table 1:** Characteristics of qualitative study participants and relationships (n = 80)^a^

Participant characteristics	AGYW (n = 49)	Male partner (n = 31)
	Mean (sd)	Mean (sd)
Age (years)	21.1 (2.1)	26.4 (4.9)

	n (%)	n (%)
Education completed		
Some primary	1 (2)	2 (6)
Some secondary	12 (24)	5 (16)
Completed secondary	20 (41)	16 (52)
Any tertiary	16 (33)	8 (26)
South African	45 (92)	20 (65)
Relationship status		
Casual	9 (18)	4 (13)
Steady, not living together	31 (64)	14 (45)
Steady, living together	9 (18)	11 (36)
Married	0 (0)	2 (6)

Characteristics of the AGYW in the relationship^b^	n (%)	n (%)
Age category of AGYW		
Adolescent girl (16‐19 years)	13 (27)	11 (35)
Young woman (20‐24 years)	73 (73)	20 (65)
AGYW is pregnant	3 (6)	11 (35)
AGYW is a mother	21 (43)	11 (35)
AGYW had more than one sexual partner in past six months	11 (22)	2 (6)
AGYW has steady income	13 (27)	8 (26)
AGYW perceives male partner to have multiple partners	15 (31)	7 (23)
AGYW perceives male partner to have more power in the relationship	28 (57)	18 (58)

AGYW, adolescent girls and young women.

a Overall, 91 individuals participated in the qualitative study. Quantitative information missing for n = 9 AGYW not enrolled in the parent study (refused to take the HIV self‐test kit, but agreed to the qualitative interview), 1 AGYW unlinked to her quantitative results, and 1 male partner, thus n = 80 for this table

b As reported by the female partner.

### 3.1 Relationship typologies

**Figure 2:**
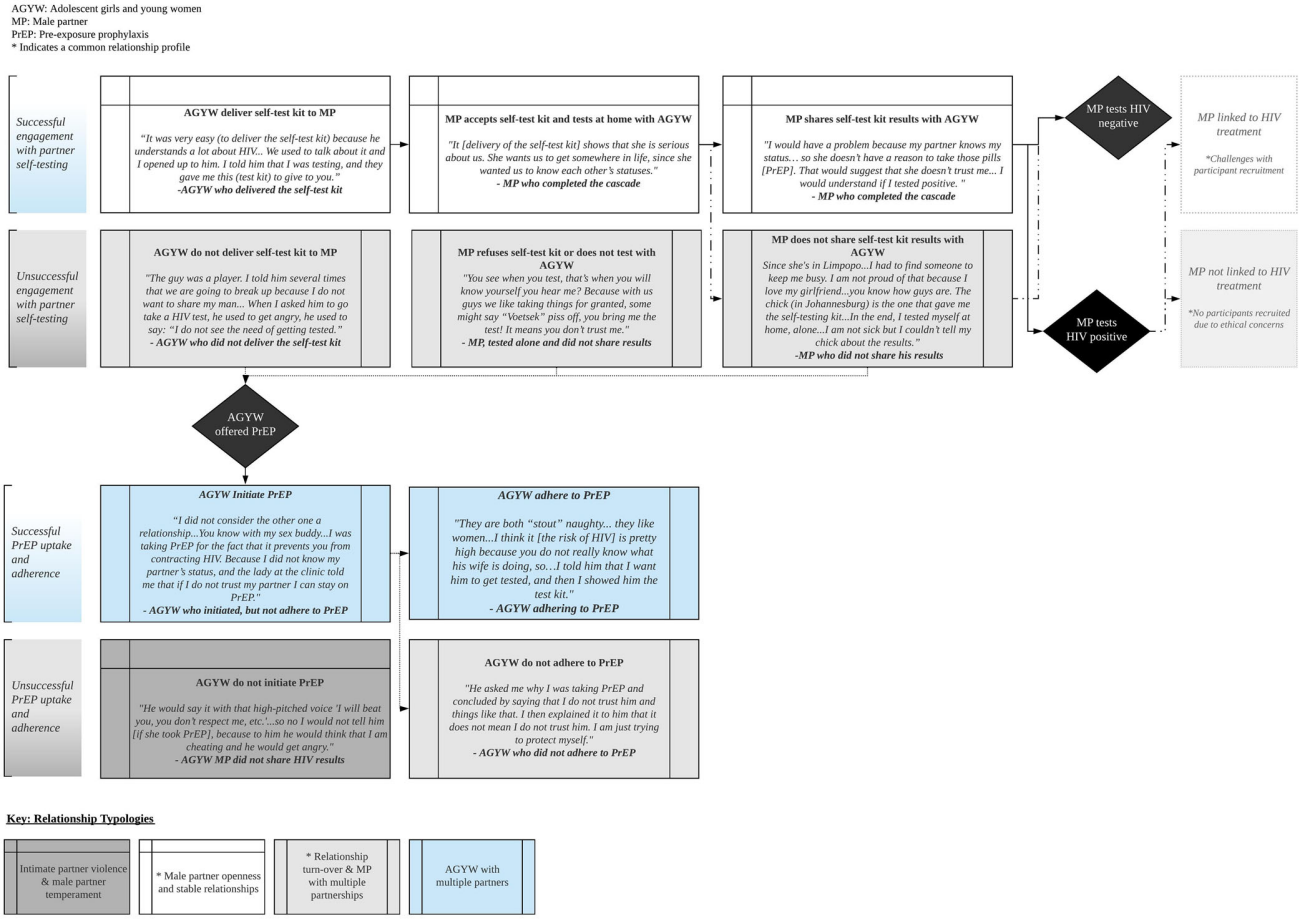
Relationship typologies and engagement in the HIV prevention cascade for self‐testing and PrEP interventions. AGYW, adolescent girls and young women; MP, male partner; PrEP, pre‐exposure prophylaxis. *Indicates a Common relationship profile.

Four relationship typologies emerged that influenced completion of the two HIV prevention cascades for AGYW and MPs. The two most common relationships were characterized by high turnover and/or multiple partnerships, mainly AGYW with MPs who held multiple partnerships, though also multiple partnerships held by an AGYW. Less common was a third type of relationship characterized by IPV. Despite screening for IPV prior to intervention enrollment, approximately 25% of the interviewed AGYW indicated they had a violent MP. The fourth and common relationship type was characterized by stability and/or the MP’s openness. Detailed relationship profile descriptions and their general impact on cascade completions are detailed below and summarized collectively in Figure 2.

#### 3.1.1 Relationship turnover and multiple partnerships

Many AGYW indicated their primary partner had relationships with other women. In these cases, AGYW were often the secondary partner of a man who was married or had a female partner living in another province. Despite the high acceptability of self‐testing demonstrated in the parent study, in almost all cases where AGYW had a MP with multiple partners, self‐test kit delivery was a challenge. AGYW did not perceive the intervention as a mechanism to facilitate discussions around HIV, but rather, thought it would invite distrust into the relationship and insinuate non‐monogamy from either the AGYW or MP.

I think he has a ‘makhwapheni’ [side chick] … When I take this kit with me, we are going to fight. He was going to take it personally: ‘you should have told me that you suspect me of something’. – AGYW who did not accept self‐test kit

In most cases when the MP was believed to have multiple partners, AGYW did not accept the intervention or accepted the self‐test kit but did not deliver it. Other AGYW in this category viewed self‐testing as an opportunity to learn their partner’s status, but when attempting to deliver the self‐test kit met resistance and/or were unsuccessful at convincing their partner to test in their presence, or never received the results. In some cases, the MP’s response to self‐testing altered AGYW perceptions of the relationship, with AGYW indicating that they trusted their partner less or feared that he might be HIV positive.

He took the test privately. When I asked him about the results, he said he tested negative. I asked him where the proof is, then he told me that there is no proof. I thought maybe he is positive. – AGYW whose MP did not share results

Despite AGYW perceiving their MP’s response to self‐testing as a sign of an HIV‐positive status, this was rarely identified as influencing her motivations to uptake PrEP. Most AGYW in this typology acknowledged PrEP as a risk reduction strategy, but few initiated it. Reasons paralleled concerns around self‐testing, with many AGYW indicating it would signal to MPs that she or the MP were non‐monogamous.

Another prominent concern was fear that MPs might misinterpret an AGYW’s intentions to take PrEP as an accusation that he is HIV positive. Similarly, for those who initiated PrEP but did not adhere, challenges around relationship trust emerged and negatively impacted adherence. For instance, most AGYW indicated they would not tell their partners they were using PrEP out of fear it would elicit an angry reaction or lead to relationship tension. Interviews with MP’s demonstrated these AGYW concerns were valid.

I would ask why does she want to take such a thing? It will definitely mean she wants to sleep around. – MP, accepted test and shared results

Furthermore, within this relationship characterization, issues of transient and long‐distance relationships emerged, notably with MP from outside Johannesburg having primary partners at home. Depending on the type of relationship in which an AGYW was involved, engagement in self‐testing fluctuated, with some AGYW unable to engage their partner in testing or results sharing despite successfully delivering the test‐kit. In most cases, AGYW indicated intention to deliver the kit but did not because they had not been with their partner since receiving the test. In other cases, AGYW would deliver the self‐test kit to their MP, but he shared his results with his primary partner, but not secondary partners. Regarding PrEP, all AGYW in relationships with transient men held the perception that their MPs likely had multiple partners but expressed little urgency around initiating PrEP.

#### 3.1.2 AGYW with multiple partners

There were a few cases where AGYW indicated they were engaging with multiple men until they were ready to be in a more serious relationship. These AGYW viewed self‐testing and PrEP as mechanisms for protecting one’s health and demonstrated confidence in accepting or delivering the self‐test kit. Almost all AGYW with multiple partners initiated PrEP because they understood the risks associated with their behaviour or the behaviours of their partners. However, AGYW did not want to disclose PrEP use to their partners due to concerns of how MP might react. For AGYW who did initiate PrEP, adherence varied – many stopped taking PrEP due to side effects.

He does not know I am on PrEP … It would come as if I do not trust him. He might think that I think that he is sleeping around with many girls. ‐ AGYW, initiated PrEP

Interviews with the MPs supported AGYW’s concerns around PrEP and self‐testing and most MPs disclosed they had multiple partners. MPs reported mixed perceptions around PrEP – some approved of PrEP as a protective intervention for AGYW but would not support it if their sexual partner used it. Others demonstrated concern that offering AGYW PrEP would increase non‐monogamy by providing AGYW with the protection and thus freedom to have sex with multiple men.

PrEP is good because it will protect her, but I have concerns about it … There are sexually active women out there having multiple partners without protection. In that situation … she might end up being a [HIV] carrier. – MP, accepted test and shared results

#### 3.1.3 IPV and male partner temperament

While AGYW in violent relationships were not the target recipient of this intervention, some AGYW indicated experiences of IPV or indicated they had a temperamental, angry partner. These AGYW were largely concentrated in the group of women who did not accept or did not deliver the self‐test kit or take PrEP. AGYW in this group universally acknowledged that PrEP and partner self‐testing would be beneficial but chose not to accept either intervention because they believed it would elicit violence.

Many AGYW explained that when they historically tried to discuss HIV or testing with their partner, he would react violently. Furthermore, a subset of AGYW who lived with their partners and were interested in PrEP expressed fear that their partner would react violently if he found the pills.

I did not deliver the self‐test kit. This guy is very crazy, and I fear him … When I asked him about the test, he became angry. People say he is abusing his wife, and I am afraid he will beat me. – AGYW who did not deliver the self‐test kit

Violent MPs were not recruited for qualitative interviews, prioritizing safety of the AGYW. However, many MP IDI participants on their own accord mentioned that neither partner self‐testing nor PrEP would be plausible interventions for women with violent partners, reinforcing AGYW concerns.

#### 3.1.4 Male partner openness and stable relationships

The self‐testing intervention was successful among AGYW who were in stable relationships, which include AGYW who described their relationships as “committed” or “serious,” who claimed they “trusted” their partner, who were in a relationship for many years, and who were only dating each other. This relationship type was the second most common sub‐study profile. The intervention was also successful for AGYW who described their partner’s temperament as “open‐minded,” “accepting” and “understanding,” and whose partners were willing to discuss HIV‐related subjects. In many of these cases, AGYW had previously discussed HIV with their partner or had previously tested together. In other cases, AGYW were confident offering the self‐test kit because they believed their MPs would be receptive to HIV discussions.

It was not disappointing because he did not make a big deal out of it. He understood that testing for HIV was a necessity and he was really open to it. – AGYW delivered self‐test kit

AGYW engaged in a serious relationship completed the cascade steps with ease. Specifically, AGYW and MPs who successfully tested together reported increased trust and openness resulting from the self‐testing intervention.

Most AGYW in stable relationships reported no need to initiate PrEP because they trusted their partner, felt confident they could communicate about testing in the future, or could convince him to use alternative prevention methods such as condoms.

I am not interested in taking PrEP because I am dating one partner and he is only dating me. – AGYW did not take PrEP

Overall, MPs in stable, serious relationships (as defined above) perceived self‐testing as a gesture that their AGYW was committed to the relationship and to protecting his health. MPs in this subgroup also held the perception that PrEP was a beneficial intervention for many women but felt that their own sexual partner should have no reason to initiate PrEP, especially if he was HIV negative.

She wasn’t forcing me but was asking me and that made me happy. My partner loves me so much that she brought me an HIV self‐test kit so that I can know where I stand. – MP, accepted test and shared HIV results

## 4 DISCUSSION

While there is extensive literature examining adolescent and young adult relationship dynamics in South Africa [4, 5, 6, 7, 8, 9], less is known about how relationship profiles impact engagement across the HIV prevention cascades for female‐initiated prevention methods. Applying the prevention cascade framework through qualitative sampling and analysis generated insights regarding relationship heterogeneity and completion of HIV prevention cascades that might not have been observed in a random sample. This may be particularly important as sensitive information not reported in the parent study, such as violence and multiple partnerships, would have gone unidentified. Use of qualitative methods in the sub‐study allowed for more nuanced insight into the challenges AGYW face completing the prevention cascades of self‐testing and PrEP interventions.

A critical insight gleaned from this analysis is that AGYW in high‐risk relationships had lower success navigating the HIV prevention cascades, even for female‐led interventions. For situations in which the MP was perceived to have multiple partners or was violent, AGYW had limited success engaging with both PrEP and secondary distribution of self‐testing interventions. Consistent with other studies highlighting the non‐monogamous and transient nature of relationships for South African AGYW, these results demonstrate that the adoption of PrEP and delivery of self‐test kits from AGYW to MPs is often impeded by the same gender norms that disadvantage AGYW in engaging with other HIV prevention options, such as negotiating male condom use [4, 5, 6, 7, 8, 9, 22, 25, 26]. Furthermore, recent studies on female PrEP use in Johannesburg have shown that a woman's ability and willingness to use PrEP is strongly influenced by her MP and that perceived or actual MP resistance makes adherence difficult [27]. Purposive sampling of AGYW and MP across completion of the two prevention cascades illuminated several of these barriers that were unelicited through the quantitative parent study and reinforce evidence that AGYW face similar perceived or actual relationship‐related barriers to HIV prevention. This further suggests that increased emphasis should be placed on screening for relationship characteristics during implementation to gauge whether MPs will support or oppose an AGYW’s involvement in HIV prevention [26, 28]. Depending on the type of relationship, implementers can encourage AGYW to disclose PrEP use to MPs, or suggest other strategies that minimize potential adverse reactions while still engaging in prevention methods where possible [28].

While the primary recipients of the intervention were not AGYW experiencing IPV or AGYW living physically distant from their partners, the cascade sampling approach revealed the pervasiveness and under‐reporting of violence in South African youth and the nuances behind long‐distance partnerships. These various relationship manifestations can hinder AGYW’s engagement in interventions they perceive as protective and beneficial [4, 5, 6, 7, 8, 9, 22]. For young women whose partners have multiple partners, recognizing her barriers to HIV prevention uptake and fully considering the relationship dynamics may be critical to support her navigation through prevention options.

Despite challenges, the interventions were successful for many AGYW who were in partnerships with MP characterized as supportive or open in their communication patterns. These AGYW viewed self‐testing and PrEP as mechanisms for empowerment and protecting one’s health, and demonstrated confidence in accepting, delivering and seeing the results of the HIV self‐test kit and initiating PrEP. This finding reinforces that test kit delivery is highly acceptable and desirable outside the ANC context [10]. Furthermore, developing positive patterns of engagement with MPs at a young age may carry forward to support AGYW’s navigation of continued HIV testing and prevention practices within relationships as she ages. While non‐adherence and discontinuation of PrEP among AGYW in sub‐Saharan Africa is well documented and inhibits PrEP effectiveness [23, 24, 26, 27, 28, 29], these results reinforce that young women’s decisions not to use PrEP are frequently rational and represent the delicate balance of weighing relationship risks and benefits [23]. Furthermore, understanding the delineation between AGYW engaged with a man who has multiple partners or AGYW engaged with multiple partners herself could help to identify whether an AGYW will perceive an intervention as an empowerment mechanism for addressing her own risk versus an intervention that threatens stability and trust within her relationship [25, 28].

Together, these findings suggest that the starting place for tailoring counselling and delivery of HIV prevention interventions for young women is not just a review of her sexual behaviour and vulnerabilities, but an assessment of her relationship environment. Strengthening relationship dynamics within intimate partnerships to prevent HIV has been previously suggested [25, 27, 29], and future work should identify how unmarried AGYW select into relationships with men who exhibit positive relationship qualities or how they develop relationships in which they can practice open communication. As an increasing number of HIV prevention programs begin to engage young men [10, 11, 12, 13], both young women and men should be counselled on basic healthy communication patterns, with online digital health and/or mentorship targeting adolescents in this space promoted [25]. Furthermore, to tailor interventions to effectively address the complexity behind HIV prevention implementation, these results emphasize the need for strategies that address violent behaviour or support young women in the removal from her violent situation [23, 25].

This study has limitations. Given that this was a qualitative study, we cannot generalize to the entire population of AGYW. Additionally, due to the parent study design, AGYW in identifiably violent relationships were not enrolled in the intervention, thus, results may not be generalizable to AGYW in more violent relationships. Furthermore, the study relied on self‐reported data from the AGYW, potentiating misreporting by AGYW about intervention completion either in the parent or qualitative studies. For instance, IPV was disclosed in the qualitative study. Additionally, AGYW and MPs agreeing to interviews may be different from those not represented, suggesting that other intervention challenges and facilitators might exist but were not captured. However, the study was able to successfully recruit AGYW from each step of the prevention cascades, apart from HIV‐positive MPs, reflecting a diverse set of participants. Finally, participants were strategically selected for qualitative interviews based on their placement in the cascades and were not representative of HIV prevention cascade completion, but rather highlight typologies of AGYW relationships that succeeded or struggled with cascade engagements.

## 5 CONCLUSIONS

Overall, applying the prevention cascade approach to qualitative sampling of AGYW enrolled in PrEP and secondary distribution self‐testing interventions reinforced the nuance behind relationship factors and HIV prevention, and suggested that screening around relationship types and differentiating HIV prevention services and counselling according to the specific relationship situations could improve intervention uptake and outcomes. To date, interventions targeting AGYW in isolation have often produced disappointing findings, as they rarely account for the context in which individuals live. Identifying and tailoring interventions to AGYW’s environments, specifically factoring in long‐distance relationships, living situations, sexual networks, and partnership dynamics may hold promise for achieving greater HIV prevention success. Furthermore, incorporation of communication skills‐building strategies within larger HIV prevention interventions for adolescents may offer benefits for HIV prevention and general well‐being.

## COMPETING INTERESTS

The authors have no competing interests to declare.

## AUTHOR’S CONTRIBUTIONS

JB, LM, AVR, SS, MK and TP designed the study. MM, LM, JB, TP, LH and SS oversaw data collection. MM, LM, LH, MK, AC, SS and TP reviewed the content of the transcripts as they came in and engaged in regular debriefing and analysis calls with the data collection team. LH and AC coded the transcripts. All authors contributed to the writing of the manuscript and have read and approved the final version.
